# Asymmetric Dialysis: Truly Unified and Simultaneous Single‐Pass Concentration and Buffer Exchange

**DOI:** 10.1002/bit.70218

**Published:** 2026-05-12

**Authors:** Ujwal Patil, Michelle Chen, Irina Ramos, Jon Coffman

**Affiliations:** ^1^ Bioprocess Technologies and Engineering Biopharmaceutical Development, AstraZeneca Gaithersburg Maryland USA

**Keywords:** asymmetric dialysis, bioprocess intensification, continuous manufacturing, counter‐current dialysis, diafiltration, single‐pass bioprocessing, ultrafiltration

## Abstract

Biopharmaceutical manufacturing has been using ultrafiltration (UF) and diafiltration (DF) for buffer exchange, desalting, and formulation of biologics. The legacy UF/DF is commonly a two‐step batch process that is challenging to integrate into end‐to‐end continuous biomanufacturing. Here, we introduce asymmetric dialysis, a novel one‐step continuous process that combines UF and DF. It works by utilizing asymmetric flow between the inlet and outlet of the retentate and complementary flow of the dialysate solution, achieving product concentration, buffer exchange, and salt removal using a commercially available hollow fiber device. Asymmetric dialysis can achieve product concentrations of 105 (3.8×), 200 (10×), and 64 g/L (9.4×) starting from feed concentrations of 30, 20, and 7 g/L, respectively, with modest pressures of 6–7 psi. The interplay between feed and exchange buffer flow rates was exploited to make the process sustainable by reducing buffer consumption by 75% (25 L/kg mAb) compared to conventional batch UF/DF (100 L/kg, mAb). We successfully processed 7 kg of mAb at 20 g/L feed using 5‐day asymmetric dialysis with a daily productivity of 0.8 kg/m^2^/day to product concentration of 200 g/L. These results demonstrate the potential of asymmetric dialysis, a simple, sustainable, and low‐cost bioprocessing technology for continuous bioprocessing.

## Graphical abstract

Asymmetric dialysis, a novel one‐step continuous process combining ultrafiltration and diafiltration, achieves high product concentrations (up to 200 g/L) with low pressures and reduces buffer consumption by 75% compared to conventional methods. This sustainable, low‐cost technology demonstrates significant potential for integration into continuous biopharmaceutical manufacturing.

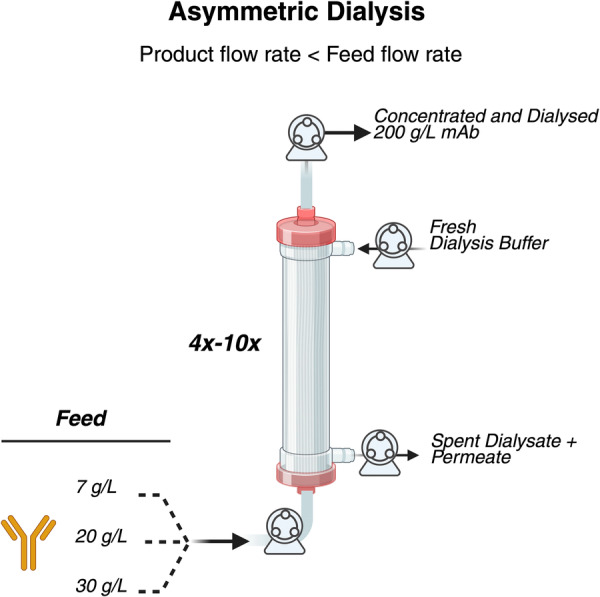

## 1 Introduction

Biopharmaceutical manufacturing relies heavily on membrane‐based processes to ensure the safety and quality of the drug products. In downstream purification, the membranes are most used for sterile filtration, virus retentive filtration, product concentration, buffer exchange, and pre‐formulation. Depending on the application, the membrane processes are operated in either dead‐end filtration (DE) or Tangential flow filtration (TFF) mode. TFF is typically operated at a relatively high flux (300 LMH) to enable high‐speed movement of feed along the plane of the membrane, in turn facilitating disruption of concentration polarization and membrane fouling. Conventionally, TFF systems such as ultrafiltration (UF) and diafiltration (DF) have been used to perform concentration and buffer exchange of biologics, viz., monoclonal antibodies (mAbs). In UF/DF, the feed solution is repeatedly cycled through a membrane module in a closed loop until the desired degree of concentration and buffer exchange are attained. The inherent batch nature of the UF/DF makes it unamenable for process integration with lower flow rates encountered within continuous manufacturing (Coffman et al. 2021, 1735).

**Figure 1:**
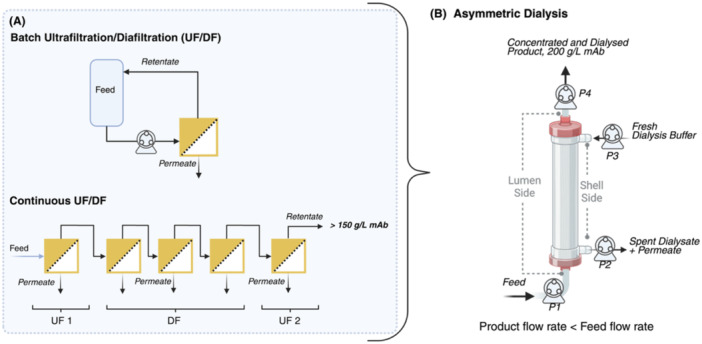
Comparison of legacy Ultrafiltration/Diafiltration (UF/DF) methods with hollow‐fiber asymmetric dialysis. Unlike batch and continuous UF/DF (A), asymmetric dialysis (B) performs simultaneous concentration and buffer exchange in a single pass. The feed containing mAb is pumped into the hollow‐fiber cartridge using pump P1. The concentration factor along the lumen side of the cartridge is modulated using pumps P2 and P4. The fresh dialysis buffer is delivered to the shell side using pump P3.

Continuous manufacturing necessitates the use of technologies that allow the forward movement of products throughout the process without any product recirculation loops. This has prompted the introduction of single‐pass tangential flow filtration (SPTFF) in membrane operations. In SPTFF, the feed is passed through the membrane module once to attain a desired concentration without product recirculation. This mode of operation relies on higher residence times obtained by using a long feed channel in combination with careful choice of feed and retentate flow rates to achieve target concentration factors compared to traditional TFF. The extended feed channel in the module is typically obtained by using a larger membrane area or adding repetitive units of identical area or deliberate (“Christmas tree”) staging of area for optimum flow channel length (Madsen et al. 2022; Yehl and Zydney 2020; Goodrich et al. 2020; Jabra et al. 2019). Continuous UF/DF with cassette‐based SPTFF involves a multi‐stage SPTFF configuration with interim dilution in co‐current or a countercurrent operation, achieving a buffer exchange upwards of 99% (Rucker‐Pezzini et al. 2018; Nambiar et al. 2018; Kurnik et al. 1995). Even though the counter‐current approach affords a lower buffer utilization, the counter‐current system is accompanied by a higher degree of operational complexity (Figure 1A). Unlike convective methods that become flux‐limited due to polarization effects, forward osmosis and diffusive approaches for protein concentration and buffer exchange have also been reported (Chen et al. 2019; Minier‐Matar et al. 2016; Tangry et al. 2025). The dialysis approach is operationally simple yet requires a pre‐concentration step to achieve efficient buffer usage (Yehl et al. 2019; El‐Okazy et al. 2025). To date, SPTFF‐based UF and DF operations have been distinctively separate and most often operated in a connected manner to make them amenable to continuous manufacturing. Recently, a proof‐of‐concept simultaneous UF and DF operation was proposed and demonstrated using a 3D‐printed SPTFF prototype; however, the system currently lacks the scalability and robustness required for practical deployment (Tan and Franzreb 2020, 2022).

In our earlier work, we discussed the design and framework for integrated and continuous biomanufacturing, highlighting the capabilities, complexity, and sophistication required for commercial implementation (Coffman et al. 2021, 1735). Here, we report the use of commercially available hollow fiber membrane cartridges as a platform for performing simultaneous one‐step continuous ultrafiltration, buffer exchange, and formulation of biotherapeutics. The innovative, truly unified technology discussed in this work, referred to as asymmetric dialysis, is capable of 10‐fold product concentration and buffer exchange in a single pass. The term “asymmetric” refers to the intentional mismatching of the entry and exit flow rates in the system and should not be confused with the characteristics of membrane pores. The adoption of a hollow‐fiber format lowers spatial requirements compared to flat‐sheet cassette configurations. We evaluated the suitability and effectiveness of various hollow fiber devices for asymmetric dialysis, along with strategies for process intensification and minimizing buffer consumption. The goal was to strike a balance between spatial economy, bioprocess readiness, ease of step development, and process performance. This work also discusses considerations for the commercial implementation of asymmetric dialysis. Our results demonstrate that asymmetric dialysis can perform simultaneous concentration and buffer exchange of monoclonal antibodies (mAbs) in truly continuous operation in a simple, sustainable, and low‐cost manner.

## 2 Materials and Methods

All experiments were performed using either Optiflux 180NR (Fresenius, USA) hemodialyzer or Minikros 0.16 m^2^ (Repligen, USA, Cat# S04‐E030‐05‐N) bioprocess cartridge. The properties of the hollow fiber cartridges used in this study are noted in Table 1. A feed containing mAb A (IgG_1_) or B (IgG_1_) was adjusted to pH 5.0 using 50 mM sodium acetate, 200 mM sodium chloride, pH 5.0 buffer (conductivity, 21 ± 2 mS/cm) to mimic the cation exchange mAb elution pool. The mAb concentration in the feed was adjusted between 7 and 30 g/L. All the experiments discussed in this work were performed with 20 mM histidine/histidine HCl, pH 5.9 ± 0.1, as the dialysis buffer.

**Table 1:** Properties of hollow fiber cartridges.

Cartridge	Manufacturer	Membrane type	Inner diameter (μm)	Wall thickness (μm)	Fiber length (cm)
Optiflux 180NR— hemodialyzer	Fresenius	Polysulfone	190	45	30
Minikros— bioprocess hollow fiber	Repligen	Polyethersulfone	500	200	25

### 2.1 Experimental Set‐up for Asymmetric Dialysis

A hollow fiber cartridge was placed in a vertical position, and the feed was introduced from the bottom into the lumen‐side port using pump P1 (as shown in Figure 1B). The shell‐side port (shell‐outlet) closest to the feed port was connected to pump P2, while the distal shell‐side port (shell‐inlet) and lumen‐side port (lumen‐outlet/retentate) were connected to pumps P3 and P4, respectively. The dialysis buffer was introduced into the shell side using pump P3, while pump P2 controlled the flow rate at the shell outlet. All the hollow‐fiber cartridges were operated in counter‐current mode. The final concentrated and buffer‐exchanged product (retentate) was collected at the lumen outlet. Before operation, the peristaltic pumps (P1, P2, P3, and P4) were calibrated by timing the collection of a known volume of deionized water using a digital balance, using the appropriate pump heads and tubing length that fit the operation.

### 2.2 Asymmetric Dialysis Operation

For a typical four‐pump setup using a 1.8 m^2^ Optiflux 180NR hemodialyzer, pump flow rates were adjusted to allow simultaneous product concentration and buffer exchange as follows; the feed flow (P1) was 20 mL/min (0.7 L/m^2^/h or 0.7 LMH) and the pump P4 was adjusted to 5 mL/min to allow retentate flow control and achieve a 4× concentration factor along the hollow fiber membrane. Pressures were monitored using sensors before and after the inlet/outlet ports. All solutions used in the experiment were filtered through a 0.22 μm PES filter (VWR, USA, Cat# 10040‐468). The shell and lumen compartments of the hollow fiber cartridge were flushed with dialysis buffer before the experiment. On the shell side, the pumps P2 and P3 were adjusted to 60 and 45 mL/min, respectively. The Optiflux 180NR hemodialyzers have a 100 mL hold‐up volume; therefore, a steady state was defined as the passage of 2.5 times the hold‐up volume through the cartridge. The product (retentate) concentration was measured after allowing the system to reach a steady state. For the experiments involving bioprocess hollow fiber, a feed flow rate was chosen to yield a feed flux that matched the hemodialyzer experiments. All experiments were performed at room temperature. The impurity removal performance of asymmetric dialysis was investigated using vitamin B_12_ (Sigma‐Aldrich USA, Cat# V2876) as a model impurity in the feed. All experiments were performed in single‐pass mode with no recirculation. Samples were collected periodically from the lumen outlet for offline pH and conductivity (Mettler Toledo, USA). Mab and B_12_ concentrations were measured using SoloVPE (Repligen, USA) at 280 and 550 nm, respectively. The histidine analysis was performed using Acclaim Trinity P1 column (ThermoFisher Scientific, USA, Cat# 075563).

### 2.3 Definition of Asymmetric Factor (AF)

For legacy UF processes, the degree to which the volume of a solution has been reduced (concentration) is indicated by the volumetric concentration factor (*vcf*), and the extent of buffer consumption in DF is expressed as diafiltration volume (DV). To succinctly denote the simultaneous concentration and buffer exchange afforded by asymmetric dialysis, we introduce the term asymmetry factor (AF) expressed as ${\mathrm{AF}}_{\alpha^{\prime }}^{\mathrm{vcf}}$. Herein, *vcf* is the volumetric concentration factor along the lumen, calculated as the ratio between feed flow and retentate flow; *α′* is the buffer consumption calculated as the ratio between dialysis buffer flow and retentate flow. For example, a process with a concentration factor of 10 and *α′* of 22.5 is depicted as ${\mathrm{AF}}_{22.5}^{10}$; for additional examples and mass balances for inputs and outputs, please refer to Supporting Information S1: Table 1.

## 3 Results and Discussion

### 3.1 Screening of Hollow Fibers

**Figure 2:**
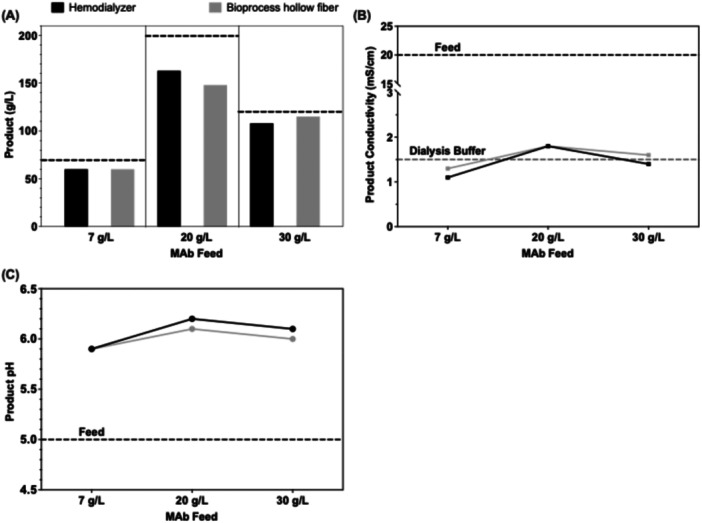
Asymmetric dialysis performance of hemodialyzer (black) and bioprocess hollow fiber (gray) cartridges. (A) Product concentrations (*y*‐axis) were obtained for respective mAb A feed concentrations (*x*‐axis). The dotted lines indicate intended target concentration factors of 10×, 10×, and 4×, for 7, 20, and 30 g/L, respectively. (B) Product conductivity and (C) pH after asymmetric dialysis for respective mAb A feed concentrations.

To determine the appropriate hollow fiber for continuous bioprocessing, the asymmetric dialysis performance was evaluated using two types of hollow fiber cartridges, (1) Optiflux F180NR (1.8 m^2^) hemodialyzer and (2) Minikros (0.16 m^2^) bioprocess hollow fiber, with mAb A feed mimicking a cation‐exchange chromatography (CEX) elution pool (50 mM sodium acetate buffer, 200 mM sodium chloride, pH 5.0). Figure 2 shows the single‐pass concentration and buffer exchange of feed containing 7, 20, and 30 g/L mAb at a feed flux of 0.7 LMH for three‐pump setup without deliberate retentate flow control (P4). Shell‐side flow rates were adjusted to target distinct asymmetry factors ${\mathrm{AF}}_{22.5}^{10}$ (for 7 and 20 g/L) and ${\mathrm{AF}}_{11.2}^{4}$ (for 30 g/L). The asymmetry factor is a practical descriptor of the degree of asymmetry in the flow rate. In operational terms, it captures the combined effect of volumetric concentration and dialysate‐to‐retentate flow relationships, both of which influence the tradeoff between buffer exchange efficiency, achievable retentate concentration, and buffer consumption. The product concentrations obtained with the hemodialyzer and bioprocess hollow fiber cartridges were comparable and yielded average product concentrations of 60, 160, and 110 g/L (Figure 2A). The hemodialyzer resulted in concentration factors of 8.5×, 8.2×, and 3.7×, whereas concentration factors of 8.5×, 7.4×, and 3.8× were achieved with bioprocess hollow‐fiber cartridges.

As shown in Figure 2B,C, both membranes were able to reduce feed conductivity from 20 mS/cm to product conductivity of 1.5 mS/cm, comparable to the dialysis buffer conductivity of 1.5 mS/cm. Similarly, for buffer exchange, the overall product pH after asymmetric dialysis was found to be 6.0 ± 0.2 for both membranes, as targeted. The transmembrane pressure (TMP) across both cartridges was less than 5.0 psi, with the highest TMP of 4.0 psi for the hemodialyzer for the product concentration of 160 g/L. Interestingly, for 7 and 20 g/L feed, both cartridges fell short of their intended concentration target of 10× by 15%. We believe that at higher (> 6×) concentration factors, the lack of flow control on the retentate limits the maximum attainable concentration factor across the cartridge. To overcome this limitation, all subsequent investigations included the use of a retentate pump that would establish enough TMP to overcome the membrane resistance, enabling consistent and tunable product concentration.

Even though both membrane units represent a general hollow fiber format, key differences in fiber dimensions, shape, packing, and shell‐side compartments could make them perform differently when operated in counter‐current flow (Table 1). For instance, the thinner fibers (~45 μm) in hemodialyzers facilitate better mass transport and are typically rated for low‐pressure (< 10 psi) operation; a characteristic recently leveraged to achieve highly selective protein separations via countercurrent diffusive transport (Yehl and Zydney 2021). In contrast, fibers for the bioprocess cartridges have higher wall thickness (~200 μm) required to sustain higher pressures (10–20 psi) in UF. In addition, the undulations on fibers in hemodialyzers from micro‐crimping contribute to enhanced mass transfer compared to straight cylindrical fibers (Ronco et al. 2000; Leypoldt et al. 2003). Unlike the bioprocess cartridges, shell‐side compartments of the hemodialyzers are equipped with an annular flow distributor or flow baffles, which mitigate the flow maldistributions on the shell‐side that can severely impact the buffer exchange performance (Noda et al. 1979; Costello et al. 1993; Ward et al. 2011; Lemanski and Lipscomb 2002; Poh et al. 2003). Historically, bioprocess hollow fibers were designed for UF, not dialysis, and hence have not benefited from the technological improvements made in hemodialyzers over the four decades (Said et al. 2021). To take advantage of cumulative advancements, we opted to utilize hemodialyzers in our subsequent studies.

### 3.2 Intensification of Asymmetric Dialysis

**Figure 3:**
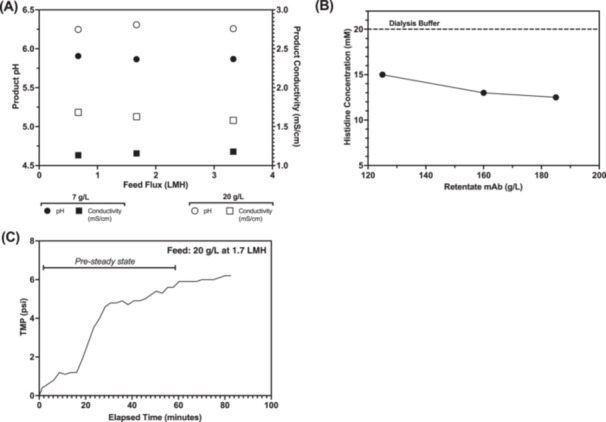
Performance of intensified asymmetric dialysis using hemodialysis cartridge (Optiflux 180NR) (A) Product pH (primary *y*‐axis, circle) and conductivity (secondary *y*‐axis, square) after asymmetric dialysis of 7 g/L (black) and 20 g/L (white) mAb feed. (B) Histidine concentration in the AD product using 20 g/L feed at various target product concentrations compared with His concentration in the dialysis buffer (dashed line, 20 mM). (C) TMP profile for 20 g/L feed at 1.7 LMH feed flux and 10× concentration factor.

To explore opportunities to intensify asymmetric dialysis for higher product throughput, we performed scouting experiments by varying feed concentration and feed flux and evaluated their impact on process performance. We examined the effect of increasing feed flux at 7 and 20 g/L mAb while holding dialysis buffer composition and asymmetry factor ${(\mathrm{AF}}_{22.5}^{10})$ constant. Process performance was assessed using achieved retentate concentration, conductivity, pH, and TMP. All the studies were performed in the retentate flow control mode using a peristaltic pump (P4) to attain the desired concentration factor. The range of feed flux evaluated 0.7–3.3 LMH represents practically relevant productivity scenarios in integrated and continuous manufacturing (Figure 3) (Coffman et al. 2021, 1735; Ramos et al. 2023).

For both 7 and 20 g/L feeds, asymmetric dialysis utilizing $AF_{22.5}^{10}$ achieved an average volumetric concentration factor (~9.6) that was near the target value of 10, for feed fluxes of 0.7 and 1.7 LMH (Table 2). However, for 20 g/L feed at 3.3 LMH, the system suffered over‐pressurization (TMP > 10 psi) while approaching the target concentration. This is likely due to critical‐flux limitations at higher feed flux exacerbated by the gel layer formation, ultimately leading to increased membrane resistance.

**Table 2:** Observed retentate concentration for 7 and 20 g/L mAb feed at increasing feed flux.

Feed mAb (g/L)	Feed flux (LMH)	Asymmetry factor (vcf; *α*′) ${\mathrm{AF}}_{\alpha^{\prime }}^{\mathrm{vcf}}$	Retentate concentration (g/L)	
			Target	Observed
7	0.7	10; 22.5	70	59
	1.7			65
	3.3			56
20	0.7		200	215
	1.7			195
	3.3			188

In parallel with concentration, the buffer exchange performance was monitored. As shown in Figure 3A, processing 7 g/L mAb feed with ${\mathrm{AF}}_{22.5}^{10}$ did not negatively impact buffer exchange performance; the final product achieved a pH and conductivity comparable to those of the 20 mM histidine dialysis buffer (pH 5.9 and 1.5 mS/cm, respectively). For 20 g/L mAb feed, product conductivity was comparable to the dialysis buffer, whereas the product pH was overshot by 0.2 pH units compared to the dialysis buffer. The dependency of pH offset on target product concentration when comparing between 7 and 20 g/L for the same ${\mathrm{AF}}_{22.5}^{10}$ can be attributed to the Donnan effect typically observed at mAb concentrations above 120 g/L (West et al. 2021). We investigated the excipient (histidine) concentration in the product compared to the dialysis buffer. From Figure 3B, it can be observed that at 120 g/L, the product contains (25% lower) 15 mM histidine compared to the dialysis buffer (20 mM histidine). As the product concentration increases from 160 to 200 g/L, the extent of the drift is less prominent, stabilizing at 13 mM histidine. The drift in targeted pH and excipient concentration relative to the dialysis buffer in asymmetric dialysis is comparable to the ones that have been reported earlier in UF/DF (West et al. 2021; Hebbi et al. 2020; Ambrožič et al. 2021). This drift is a product of the Donnan and volume exclusion effects due to unequal partitioning of charged solutes across the membrane, leading to unequal distribution of electrolytes (Abel et al. 2018; Bolton et al. 2011). The drift in the product histidine can be compensated by increasing the histidine concentration in the dialysis buffer, like the strategy commonly employed in UF/DF (Miao et al. 2009).

As shown in Figure 3B, asymmetric dialysis demonstrated a steady‐state TMP of ~6 psi without over‐pressurization (> 10 psi) at a peak flux of 1.7 LMH. The steady‐state TMP values for 7 g/L feed ranged from 2 to 3 psi and 5 to 7 psi for 20 g/L. Overall, the steady‐state TMP for feed fluxes 0.7 and 1.7 LMH remained consistent (±10%) throughout the operation. The benign TMPs observed in asymmetric dialysis eliminate the need for engineered provisions such as stainless‐steel manifolds and heavy‐duty pumps typically employed in inherently high‐pressure (25 psi) UF/DF and SPTFF operations. This is likely due to inherent flow slippage in peristatic pumps that allows the system to self‐balance. The increasing feed flux investigation demonstrates the ability of asymmetric dialysis to process 1.5 kg mAb per (1.8 m^2^) membrane unit per day at 1.7 LMH feed flux for 20 g/L mAb feed and 200 g/L product. In addition, the tunability between 0.7 to 1.7 LMH is beneficial for accommodating changes in material throughput in an end‐to‐end continuous process and can be particularly useful in the event of process upsets.

For high‐concentration formulations, it is common for a UF/DF process to be accompanied by a secondary UF to achieve the target concentration (> 200 g/L) that can significantly increase both cost and process complexity. In addition, UF/DF necessitates feed flow tuning to counteract the flux challenges at elevated product viscosity and pressures that could exacerbate risks of protein aggregation due to shear stress and recurrent pump circulations. Owing to its low feed flux and single‐pass mode, asymmetric dialysis is a low‐shear process and can tolerate retentate viscosities up to 120 cP for high‐concentration formulations without a deliberate process and buffer optimization (Supporting Information S1: Figure 2). The ability to process high‐concentration viscous products presents an additional benefit in reducing Scope 3 emissions from bulk‐liquid freight to fill‐finish sites.

The changes in membrane performance were assessed using normalized water permeability (NWP) to measure the degree of fouling when compared with an unused membrane. The membranes typically retained 70% NWP after use (Supporting Information S1: Figure 1).

### 3.3 Reducing Buffer Consumption

The relationship between the dialysis buffer and retentate flow rate was exploited to make asymmetric dialysis more sustainable by lowering the buffer consumption. Typical dialysis uses a factor, “*α*” which is the ratio of the inlet flow rates (Yehl et al. 2019). For asymmetric dialysis, we have found that the ratio of outlet product flow rate (*q*_*P*_ *)* and inlet dialysis buffer flow rate (*q*_*D*_) is more predictive of dialysis efficiency. The buffer utilization (*α*′) in asymmetric dialysis is given as

$$\alpha \prime =\frac{q_{D}}{q_{P}}$$

$$q_{P}=q_{F}/CF.$$

The process mass intensity (PMI) is the volume of buffer consumed per kilogram of the product processed can be given as

$$\text{PMI}\;(L/\text{kg})=\frac{q_{D}}{q_{P}\times C_{p}}$$

where *q*_*F*_, feed flow; *CF*, concentration factor; *C*_*p*_, product concentration at retentate (kg/L).

**Figure 4:**
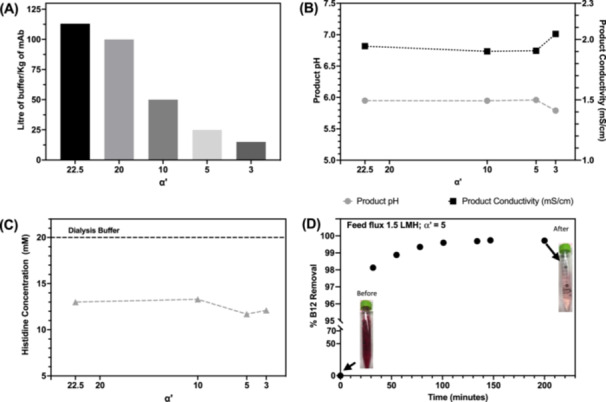
Effect of decreasing *α*′ values on buffer usage and exchange performance at 1.5 LMH feed flux. (A) Buffer consumption per kg of mAb (*y*‐axis) for a 20 g/L mAb A feed processed using asymmetric dialysis to target product concentration of 200 g/L at various *α*′. (B) Product pH (solid gray circles, primary *y*‐axis) and conductivity (solid black squares, secondary *y*‐axis) after asymmetric dialysis of 20 g/L feed at various *α*′. (C) Histidine concentration in the product after asymmetric dialysis at various *α*′. (D) Vitamin B_12_ concentration in the 225 g/L asymmetric dialysis product stream using 1.8 m^2^ hemodialyzer operated at 1.5 LMH feed containing 21 g/L mAb with 4.9 g/L vitamin B_12_ processed with ${\mathrm{AF}}_{5}^{10}$.

Figure 4A shows the buffer consumption corresponding to AF values ranging from ${\mathrm{AF}}_{22.5}^{10}$ to ${\mathrm{AF}}_{3}^{10}$, where the *vcf* is held constant at 10 and *α*′ is varied between 3 and 22.5, with 20 g/L mAb A feed at 1.5 LMH feed flux, dialyzed with 20 mM histidine buffer, pH 6.0. For an *α*′ of 22.5, the resulting dialysis buffer consumption was 110 L/kg of mAb, which is as buffer‐intensive as legacy UF/DF. In comparison, optimized *α*′ values of 5 and 3 demonstrated buffer consumption of 25 and 15 L/kg mAb, respectively. As shown in Figure 4B, reducing *α*′ to as low as 5 did not affect the buffer exchange performance with no noticeable difference in product pH and conductivity for *α*′ ranging between 22.5 and 5. Further attempts to lower buffer utilization, *α*′ = 3, resulted in poor buffer exchange performance. This indicates the possibility of using a lower *α*′ (< 3) for applications such as intermediate pH or conductivity conditioning between unit operations that do not necessitate 99% exchange. Interestingly, lowering the *α*′ beyond 22.5 had a negligible impact (10%) on product histidine concentration (Figure 4C). This indicates that the buffer exchange performance is governed by the Donnan equilibrium and is largely unaffected by the availability of the fresh dialysis buffer.

The effect of the reduced *α*′ on impurity removal was investigated by spiking the (20 g/L) mAb feed with 4.9 g/L vitamin B_12_ as a model impurity. Figure 4D shows the degree of vitamin B_12_ removal at *α*′ of 5 and 1.5 LMH feed flux. As the system reached a steady state (~70 min), the vitamin B_12_ in the product was reduced to 0.015 g/L, indicating a 99.7% removal with no impact on buffer exchange and salt removal (Supporting Information S1: Table 2). The asymmetric dialysis, at *α*′ = 5, uses 75% less buffer compared to batch UF/DF (performed at 60 g/L) and 70% less buffer than continuous cc‐SPTFF (Nambiar et al. 2018). In addition, cc‐SPTFF has an elaborate engineering design, necessitating break‐vessels and complex automation, while asymmetric dialysis is more straightforward to establish and simpler to automate. Interestingly, the estimated buffer consumption in asymmetric dialysis reduces proportionally to the increased concentration factor (Supporting Information S1: Figure 3). However, higher concentration factors for 20 g/L mab feed or higher would likely require corresponding adjustment in flux to limit system overpressurization (> 10 psi) while still maintaining the buffer savings. Overall, the results demonstrate that asymmetric dialysis is a more efficient alternative to legacy and recent technologies for concentration and buffer exchange.

### 3.4 Continuous 5‐Day Asymmetric Dialysis

**Figure 5:**
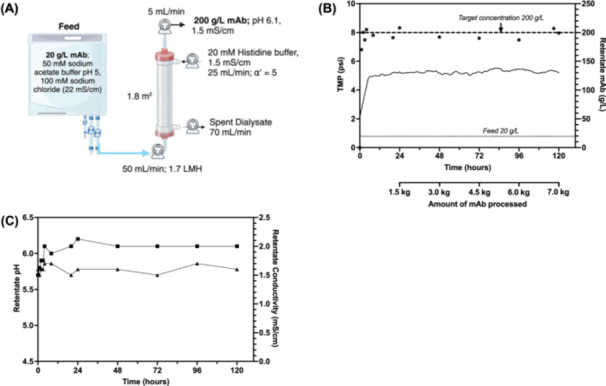
Continuous 120‐h (5‐day) asymmetric dialysis operated at 1.7 LMH feed flux. (A) Schematic of the setup with the flow rates used for lumen and shell‐side pumps to process 20 g/L mAb A feed containing 50 mM sodium acetate buffer, and 200 mM sodium chloride at pH 5 using a 1.8 m^2^ hemodialyzer cartridge. (B) Retentate mAb concentration (secondary *y*‐axis) and moving average of TMP (primary *y*‐axis) and profile over 5 days. Post‐steady state (0–2 h), the pressure profile remains largely unchanged with an average TMP of 5 psi. The retentate mAb concentrations obtained ranged between 190 and 208 g/L, well within an intended target concentration range of 190–210 g/L (gray rectangle). The floating x‐axis indicates the amount of mAb processed over 5 days with daily productivity of 1.5 kg/day. (C) pH (square) and conductivity (triangle) profile of the product throughout the 5‐day run.

Based on the flux and buffer consumption (*α*′) parameters identified above, we evaluated the endurance of the asymmetric dialysis technology for a continuous and uninterrupted operation at a feed flux of 1.7 LMH and *α*′ of 5. As shown in Figure 5A, feed containing 20 g/L mAb A, 50 mM sodium acetate buffer, and 200 mM sodium chloride at pH 5 was processed through 1.8 m^2^ hemodialyzer with a ${\mathrm{AF}}_{5}^{10}$ to yield a product concentration of 200 g/L. As demonstrated in Figure 5B, the product concentration measured at the retentate remained largely within 5% of the desired target, 200 g/L, with a measured viscosity of 21 cP. Similarly, the pH and conductivity of the product were maintained at 6.1 ± 0.1 and 1.6 ± 0.1 mS/cm throughout the 5‐day run (Figure 5C). The process operated continuously without requiring intermittent chemical or physical cleaning and flux alterations throughout the operation. Over 120 h, asymmetric dialysis processed 7 kg mAb with membrane loading of ~4 kg/m^2^. The membrane retained 60% of the NWP when compared to the unused membrane, indicating the potential to utilize the membrane beyond 5 days. Also, indicated by the constant TMP of 5 psi observed from steady‐state through the 5 days of operation. The process demonstrated an overall product recovery of 99%. Unlike UF/DF, the continuous nature of asymmetric dialysis eliminates the need for product chase; in turn, product losses for the runtime of 24 h or higher are about 1%. To note, the process was operated without any automation or human intervention after attaining a steady state, which highlights the ease of use in a lab‐scale environment.

### 3.5 Considerations for Implementation of Asymmetric Dialysis

The path to implementation of any new technology is governed by technical feasibility, robustness, integration complexity, regulatory compliance, and end‐user acceptance. In the technology life cycle, from idea to adoption, these factors evolve through stages of development, testing, and validation. Technology readiness levels provide a structured framework to assess and communicate the maturity and readiness of a technology for commercial deployment (Kedia et al. 2022). For asymmetric dialysis, we evaluated the bioprocess readiness level tool created by NIIMBL and scored up to BRL5. While it is beyond the scope of this manuscript to holistically address the requirements for implementing this technology, we aim to highlight key actions that would accelerate its successful adoption.

The process economic considerations of legacy UF/DF, SPTFF, and asymmetric dialysis are juxtaposed in Table 3 for the respective target product concentrations. Amongst the three technologies, hemodialyzers used in asymmetric dialysis offer extremely low cost of goods manufacturing (CoGm) at $15/kg, mAb. These cost savings are even more impactful towards clinical manufacturing campaigns, lowering the overall cost of drug development. Whereas, to improve the CoGm of the TFF membranes, they would need to be reused 100 times. However, each reuse incurs additional costs for labor, CIP, validation, and energy. Although theoretically feasible, this approach is not economically practical. The hemodialyzers offer a “plug and play” design for single‐use and are provided in a pre‐sterilized format, which would greatly facilitate the rapid replacement of cartridges as required. In addition, the design and simplicity of asymmetric dialysis allow a compact setup yielding a smaller GMP footprint combined with space savings due to the reduction in buffer storage area compared to legacy UF/DF (Supporting Information S1: Figure 4).

**Table 3:** Process economic comparisons for the UF/DF, cc‐SPTFF, and asymmetric dialysis.

Technology platform	Target product concentration	Buffer consumption, L/kg mAb	Sterilization/CIP	Membrane cost, $/m^2^	Protein load (kg/m^2^)	CoGm (~$/kg)
UF/DF	60 g/L	110	CIP	8000	1.4	5700
cc‐SPTFF^a^	200 g/L	60	CIP	40,000	6	6700
Asymmetric dialysis	200 g/L	25	Gamma/e‐beam irradiated	50	4 (5‐day)	15

^a^ For four‐stage cc‐SPTFF process at 10 LMH feed flux and 20 g/L feed.

Hemodialyzers, while economically attractive, require accommodations to be assimilated for bioprocess use. These cartridges do not employ conventional bioprocess connectors. This challenge can be mitigated by custom flow kits that can be assembled aseptically with membrane units, as a bridging solution until bioprocess‐ready units become available. Millions of hemodialyzers are produced globally every year, of which the biopharma sector will be a relatively minor consumer. Although this ensures a consistent supply, hemodialysis devices are classified as FDA Class II products, and their transition to bioprocessing requires appropriate certifications. For example, bioprocess vendors usually offer extractable and leachable evaluations, and the lack of bioprocessing‐ready units necessitates the creation of supporting documentation based on the available USP and BioPhorum Operations Group (BPOG) guidelines (Ding et al. 2014).

**Figure 6:**
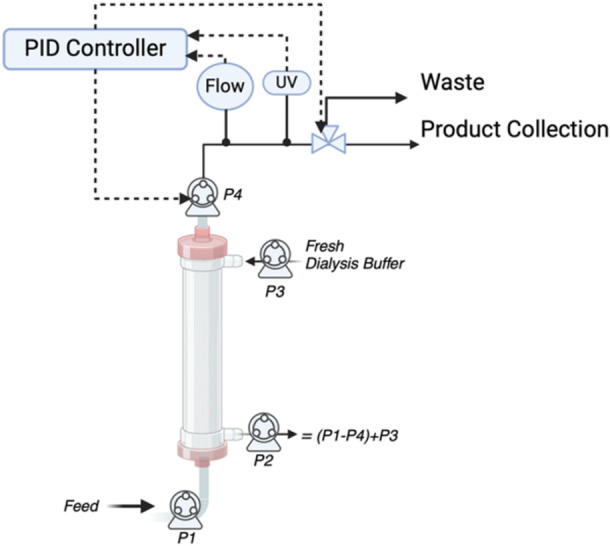
Schematic for the flow ratio‐based feedback control of asymmetric dialysis indicated by dashed arrows.

The control strategy for asymmetric dialysis is anchored in flow ratio control. The flow ratio control of the pumps is informed by continuously monitoring process parameters: flow rate, protein concentration, and TMP. The retentate pump (P4) can serve as the concentration controller to attain a consistent product concentration (Figure 6). This can be achieved by feedback control of the P4 and *q*_*P*_, measured in real‐time using an inline mass flow meter downstream of P4. During startup, until the system is in a steady state, the flow diversion valve will divert the retentate flow to waste. Once the UV setpoint is reached, the retentate flow is diverted to the product collection vessel. We believe this type of control is much simpler than the multistage TFF approaches discussed elsewhere (Malladi et al. 2023). Depending on the material accumulation rate from the preceding operation, the asymmetric dialysis feed flow can be adjusted between 0.1 and 1.7 LMH, which then dynamically adjusts the P4 and P3 to maintain the desired ${\mathrm{AF}}_{\alpha^{\prime }}^{\mathrm{vcf}}$. The spent dialysate flow P2 is set to totalize the net outflow from the system. The feed flux modulation allows the system to respond to process upsets or delays in the preceding operation and maintain a desired volume level in the feed tank.

The currently available hemodialyzers would provide a more than adequate capacity (0.8 kg/m^2^/day) for typical clinical‐scale end‐to‐end continuous manufacturing involving a 1000 L perfusion bioreactor. However, for commercial‐scale, larger membrane units need to be developed as the existing hemodialyzer membrane areas are scaled to human use with 2.5 m^2^ being the maximum. This limitation can be addressed using a scale‐out approach by operating multiple hemodialyzers either in series or parallel mode. The scale‐out approach could also open opportunities for asymmetric dialysis to be employed in fed‐batch operations where a higher membrane area is required over a shorter processing time.

## 4 Conclusions

In this work, we proposed a novel technology that can replace UF/DF operation in bioprocessing. We demonstrated practically relevant capabilities of asymmetric dialysis for continuous unified concentration and buffer exchange using commercially available hemodialyzers. The intensified process can operate at the highest feed flux of 1.7 LMH with mAb productivity of 0.8 kg/m^2^/day with minimal membrane fouling. Unlike UF/DF, where 10–100 pump passes are required, the single‐pass operation in our approach could result in improvements in product quality, especially for shear‐sensitive products. We successfully processed 7 kg of mAb with consistent salt removal and buffer exchange performance while maintaining a product concentration in a narrow range (within 5% of target) for 5 days of continuous operation. Additional engineering hardware, PATs, and automation will be required to make the technology GMP‐ready. In addition, we lowered the buffer consumption to 25 L/day, which is a 75% reduction compared to UF/DF.

Hemodialyzers offer a single‐use solution that is extremely cost‐effective ($15/kg, mAb) and with a membrane capacity upwards of 4 kg/m^2^. We have highlighted ﻿microstructural (fiber thickness) and macrostructural (cartridge design) properties of the hemodialyzers that are lacking from the bioprocess hollow fibers in the market. The hemodialyzers can be qualified for bioprocess use as a bridging solution until the bioprocess equivalent alternatives with the desired properties become available.

To summarize, the simplicity and sustainability benefits presented by asymmetric dialysis can be materialized through a concerted change management effort by addressing the technical, engineering, and regulatory nuances. We envision this technology to be forward and backward compatible, as it can be applied to continuous as well as legacy batch operations by scaling out for appropriate membrane areas. Beyond mAbs, our generalized approach can be applied to modalities such as fusion molecules, enzymes, nanoparticles, etc. Besides the advantages, opportunities for further improvements, such as the need for small‐scale down models to facilitate process development efforts, were also identified. To our knowledge, this is the first technology that achieves single‐pass simultaneous product concentration and buffer exchange in a continuous format. We believe that the benefits and simplicity of asymmetric dialysis make it a successor to state‐of‐the‐art UF/DF technologies and a key element in end‐to‐end continuous biomanufacturing.

## Author Contributions

**Ujwal Patil:** conceptualization, methodology, investigation, validation, visualization, data curation, writing – original draft. **Michelle Chen:** investigation, data curation, writing – review and editing. **Irina Ramos:** supervision, resources, writing – review and editing. **Jon Coffman:** supervision, resources, writing – review.

## Conflicts of Interest

The authors declare that some authors are named inventors on intellectual property filings, which may cover the work reported here.

## Supporting information

Supporting File

## Data Availability

Research data are not shared.
