# Supplementary material for: Asymmetric Dialysis: Truly Unified and Simultaneous Single‐Pass Concentration and Buffer Exchange
Source: Biotechnol Bioeng. 2026 May 12;123(7):1814–24. doi: 10.1002/bit.70218 (PMC13309246; doi:10.1002/bit.70218)
Supplement: Supplementary file 1 — Supporting File [file BIT-123-1814-s001.docx]

# **Supplementary Information**

**Asymmetric Dialysis: Truly unified single-pass ultrafiltration and buffer exchange**

**Ujwal Patil^*,1^, Michelle Chen^1^, Irina Ramos^1^, Jon Coffman^1^**

**^1^** Bioprocess Technologies and Engineering, Biopharmaceutical Development, AstraZeneca, Gaithersburg, MD, USA

# **Materials and Methods**

**SI Table 1**. Examples of flow rate parameters for a typical asymmetric dialysis setup with 1.8 m^2^ hemodialyzer

| **Feed Flow (P1, mL/min)** | **Shell-outlet (P2, mL/min)** | **Shell-inlet (P3, mL/min)** | **Lumen-outlet (P4, mL/min)** | **Asymmetry Factor,** $\boldsymbol{AF}_{\boldsymbol{\alpha'}}^{\boldsymbol{vcf}}$ **(vcf;α')** |
| --- | --- | --- | --- | --- |
| 20 | 60 | 45 | 5 | 4;9 |
| 25 | 76.25 | 56.25 | 5 | 5;11.25 |
| 45 | 141.75 | 101.25 | 4.5 | 10;22.5 |

# **Results**

SI Figure 1: Pre-use normalized water permeability (NWP) (legend) compared with post-use NWP of a 1.8m^2^ hemodialyzer used for asymmetric dialysis of 20 g/L mAb A feed containing 50 mM sodium acetate buffer pH 5, 100 mM sodium chloride at 1.7 LMH feed flux with AF value 10|5 for retentate mAb concentration of 200 g/L. Post asymmetric dialysis the hemodialyzer retained 68% of the NWP.

SI Figure 2: Viscosity profile for mAbs (IgG1) A (circle, grey) and B (square, solid black) in 20 mM histidine at increasing mAb concentration. The data points with red borders indicate the retentate product concentration achieved using asymmetric dialysis for 20 g/L feed at 1.3 LMH for mAbs A and B.


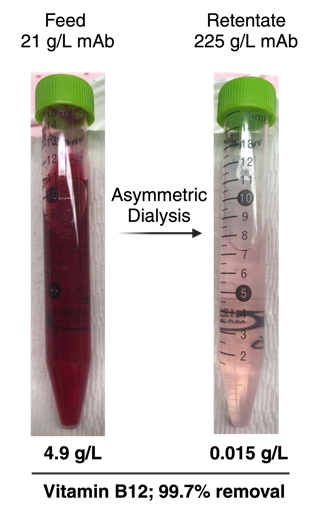
**SI Table 2**. Removal of vitamin B12 (model impurity) from mAb A feed using asymmetric dialysis

|  | **mAb (g/L)** | **Vitamin B12 (g/L)** | **Histidine (mM)** | **pH** | **Conductivity (mS/cm)** |
| --- | --- | --- | --- | --- | --- |
| Buffer | - | - | 31.0 | 5.7 | 1.8 |
| Feed | 21 | 4.9 | 0 | 5.0 | 27.1 |
| Retentate | 225 | 0.015 | 22.0 | 6.1 | 1.9 |

SI Figure 3: Relationship between buffer consumption and volumetric concentration factor (vcf). The estimated buffer requirement (L/kg of mAb) for a 7 g/L feed processed using asymmetric dialysis at feed flux of 1.3 LMH at increasing vcf.


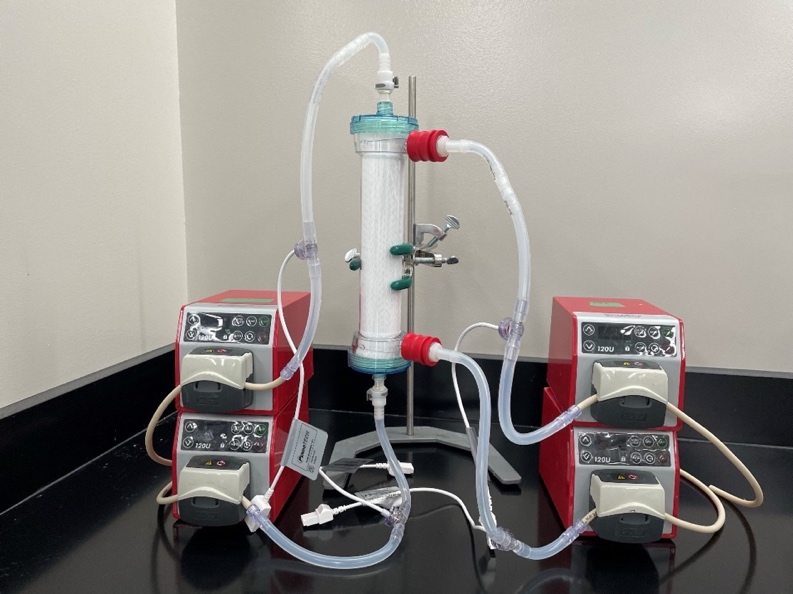


SI Figure 4: Footprint for the 2 kg/day asymmetric dialysis setup
